# Enhanced antibiotic release and biocompatibility with simultaneous addition of N-acetylcysteine and vancomycin to bone cement: a potential replacement for high-dose antibiotic-loaded bone cement

**DOI:** 10.1186/s13018-025-05637-y

**Published:** 2025-03-06

**Authors:** Tzu-Hao Tseng, Chih-Hao Chang, Chien-Lin Chen, Hongsen Chiang, Jyh-Horng Wang, Tai-Horng Young

**Affiliations:** 1 Department of Orthopaedic Surgery, National Taiwan University Hospital, 7 Chungsan South Road, Taipei City, 10002 Taiwan; 2 Department of Biomedical Engineering, College of Medicine, National Taiwan University, No.1 Jen Ai road section 1, Taipei City, 10002 Taiwan; 3 Department of Orthopaedic Surgery, National Taiwan University Hospital Jin-Shan Branch, New Taipei City, Taiwan; 4 Department of Biomedical Engineering, National Taiwan University Hospital, Taipei City, Taiwan

**Keywords:** Antibiotic-loaded bone cement, Vancomycin, N-acetylcysteine, PMMA, Cytotoxicity

## Abstract

**Background:**

Antibiotic-loaded bone cement (ALBC) is crucial for treating orthopedic infections, but its use is limited by suboptimal antibiotic release patterns and potential toxicity. This study explores the dual addition of N-acetylcysteine (NAC) and vancomycin to polymethylmethacrylate (PMMA) as a strategy to enhance the antibacterial efficacy and reduce toxicity.

**Methods:**

PMMA cement cylinders were loaded with varying combinations of NAC and vancomycin and tested for antibiotic release, cytotoxicity, and antibacterial activity over a 35-day period. Porosity of the cements was also evaluated as a measure of potential antibiotic release enhancement.

**Results:**

The addition of NAC improved vancomycin release, particularly after the initial burst release phase, and reduced cytotoxicity compared to high-dose vancomycin alone. The optimal combination was found to be 2 gm vancomycin with either 2 gm or 4 gm of NAC, which maintained effective antibacterial activity over 35 days without the toxicity seen with higher doses of vancomycin alone. Moreover, NAC alone did not demonstrate antibacterial properties, indicating its role primarily as a bioenhancer in this context.

**Conclusion:**

Simultaneous inclusion of NAC and vancomycin in PMMA bone cement provides a more favorable release profile and biocompatibility than high-dose vancomycin alone, suggesting a potential strategy for enhancing the therapeutic efficacy of ALBC in treating prosthetic joint infections. This approach allows for lower doses of antibiotics, reducing potential cytotoxicity, systemic toxicity and enhancing the duration of antibacterial activity.

**Level of evidence:**

Laboratory study.

## Background

Antibiotic-loaded bone cement (ALBC) has been used to treat orthopedic infections such as prosthetic joint infection (PJI) [1–3], infected non-union [4], and chronic osteomyelitis [5]. Effectively treating these infections is crucial as they can lead to severe complications, prolonged hospital stays, and increased healthcare costs. In the recommended two-stage surgical treatment for PJI, the first stage involves hardware removal, bone resection, synovectomy, and thorough surgical debridement, followed by the implantation of high-dose antibiotic-loaded polymethylmethacrylate (PMMA) cement beads or spacers [6]. The PMMA cement serves as a local antibiotic carrier, achieving concentrations that exceed the minimal inhibitory levels, and is typically left in place for 6–8 weeks before the final prosthesis or internal fixators are implanted [7]. This method enhances local antibiotic delivery and bioavailability, providing effective treatment.

Despite the effectiveness of this approach, the success rate of a two-stage revision for PJI varies widely, ranging from below 70–100%, depending on the patient group and the definition of “successful treatment.“ [8] The potential drawbacks of using ALBC include toxicity from excessive antibiotic release in the early stages of treatment [9], cytotoxicity from the free radicals generated during polymerization [10, 11], and a significant reduction in antibiotic release in the later stages [9, 12].

To overcome these issues, recent research has explored the incorporation of N-acetylcysteine (NAC) into bone cement. NAC, a thiol-containing mucolytic agent and a precursor to L-cysteine and glutathione, has been widely used for decades to manage respiratory conditions by breaking down mucus and improving clearance in lung disorders [13]. It was also identified as an antidote for acetaminophen overdose after the discovery that a reactive acetaminophen metabolite depletes glutathione [14]. In addition to its well-established medical applications, NAC exhibits broad-spectrum activity against planktonic bacteria and biofilms and has been shown to reduce the cytotoxicity of bone cement [11, 15–17]. Initial studies indicate that NAC-loaded PMMA is effective against biofilms and reduces cytotoxicity at both one day and one week [15, 18]. However, its antibacterial effects beyond one week remain unexplored, which is a critical limitation given that one week is insufficient for treating PJI [6, 7].

To determine whether incorporating NAC into PMMA cement truly has the potential to treat PJI, this study aims to confirm the following: (1) Whether simply adding NAC to PMMA can produce an antibacterial effect and the duration of this effect, (2) Whether the combination of NAC and antibiotics can enhance the effectiveness of ALBC and reduce its cytotoxicity, and (3) If the combination of NAC and antibiotics enhances the effect, how it compares to traditional high-dose ALBC and what the most suitable dosage would be.

## Materials and methods

### Fabrication of NAC- and/or antibiotic-loaded PMMA

Cement cylinders were created from a blend of conventional PMMA, NAC (Sigma-Aldrich, St Louis, MO), and/or Vancomycin (Sigma-Aldrich, St Louis, MO). Stryker Surgical Simplex P and Zimmer High-Fatique bone cement, two widely used commercial bone cement available in Taiwan, were used. Vancomycin was selected as the antibiotic due to its frequent clinical use and its effectiveness against methicillin-resistant Staphylococcus aureus (MRSA), a common pathogen responsible for osteomyelitis and implant-related infections [19, 20].

Referring to the commonly used high-dose ALBC dosage [6], a total of six different cement groups were prepared: (1) 2 g vancomycin in PMMA, (2) 4 g vancomycin in PMMA, (3) 2 g vancomycin + 2 g NAC in PMMA, (4) 2 g vancomycin + 4 g NAC in PMMA, (5) 2 g vancomycin + 6 g NAC in PMMA, (6) 6 g NAC in PMMA. In brief, we added the corresponding doses of vancomycin and/or NAC to 40 g of PMMA powder. The mixed powders were hand-stirred for 2 min before adding the methylmethacrylate liquid. Clindrical specimens with the size 12.0 ± 0.1 mm length and 6.0 ± 0.1 mm diameter were then created using silicone molds.

Six PMMA cylinders in each group were placed in a 50-mL flask with 10 mL of sterile phosphate-buffered saline (PBS), a volume sufficient to completely submerge both beads. The samples were then incubated at a constant temperature of 37 °C for 35 days. During this period, the PBS was completely removed and replaced at specific intervals to facilitate continuous monitoring of the elution process. The schedule for PBS removal was as follows: every 24 h for the initial 3 days, every 7 days from the 7th day to 35th day of the study.

### NAC/vancomycin elution profile

Both vancomycin and NAC were subjected to full wavelength scanning using a UV-visible spectrophotometer. Vancomycin exhibited a major peak at a wavelength of 206 nm, with a minor peak at 280 nm, while NAC exhibited a single major peak at a wavelength of 206 nm without the presence of a minor peak. The absorbance values of NAC in the concentration of 10 mg/L to 1000 mg/L at a wavelength of 280 nm are from 0.015 to 0.018. It was determined that these values do not affect the concentration measurement of vancomycin. Based on these verification results, we used UV-visible spectrophotometry to determine the concentration of vancomycin in the test substance, without considering the interference of N-Acetyl-L-cysteine.

We prepared different concentrations of vancomycin and established a calibration curve using the absorbance values at a wavelength of 280 nm. The R-squared (R^2^) value for the calibration curve is 0.9995. Since the R^2^ value is greater than 0.95, it is determined that this calibration curve is accurate and effective. After measuring the absorbance of the test substance at 280 nm, these values were substituted into the equation of calibration curve to calculate the concentration of vancomycin in the test substance.

Since the major peaks of NAC overlap with that of vancomycin in the full wavelength scanning, it is not possible to perform quantitative analysis using a UV-visible spectrophotometer. In this test for concentration of NAC, high-performance liquid chromatography (HPLC) was used for further analysis. According to the results of HPLC analysis, NAC shows a peak at 2.79 min that is suitable for analysis. Although vancomycin possesses a peak at the same time, after subtracting the peak area of the blank, peak area of vancomycin is only 49.59. Therefore, it was determined that this value does not affect the concentration measurement of N-Acetyl-L-cysteine. Based on these verification results, we used HPLC to determine the concentration of N-Acetyl-L-cysteine in the test substance, without considering the interference of vancomycin hydrochloride. We prepared different concentrations of reference NAC and created a calibration curve using the peak areas at 2.72 to 2.90 min. The R-squared (R^2^) value for the calibration curve is 0.9966, which is greater than 0.95, indicating that this calibration curve is accurate and effective.

### In vitro cytotoxicity assay

The MG-63 human osteosarcoma cell line (sourced from the European Collection of Authenticated Cell Culture, ECACC code: 86,051,601) was employed to assess cell viability. The process involved conducting a CCK-8 assay to evaluate the proliferation of MG-63 cells exposed to various extracts. Initially, cells were plated in 96-well plates at a density of 2,000 cells per well, using 100 μL of fresh medium. After a period of 24 h, these cells were subjected to different extract treatments (PBS on 1st, 7th, 21st, 35th day) for 24 h. Subsequently, 10 μL of CCK-8 solution (TargetMol Chemicals, USA) was introduced to each well, followed by an incubation period of 2 h at 37 °C. The absorbance at 450 nm was then quantified utilizing a UV–vis spectrophotometer, specifically the SpectraMax Plus-384 model (Molecular Devices, Sunnyvale, CA, USA). The results were presented as optical density values, adjusted by subtracting the absorbance of the blank wells.

### Bioassay of antibiotic activity

The antimicrobial potency of PBS from PMMA immersion collected on days 1, 2, 3, 7, 14, 21, and 35 was assessed using an agar disk diffusion assay. In short, 10-mm antibiotic discs were infused with 100μL of the PBS samples. These discs were then positioned on Mueller-Hinton agar plates (Sigma, St. Louis, MO, USA) that had been inoculated with either methicillin-sensitive Staphylococcus aureus (MSSA) or MRSA and incubated at 37 °C. The zone of inhibition (ZOI) was measured after 24 h to evaluate and compare the antimicrobial efficacy across the different groups.

### Evaluation of porosity of cement cylinders

We used the fluid displacement method to compare the porosity between different groups [21]. First, we measured the dry weight (Wa) of the PMMA cylinder. Then, we immersed the PMMA cylinder in a beaker filled with distilled water and vacuum the beaker to allow the distilled water to enter the pores. Next, we measured the weight of the PMMA cylinder (Wb). The porosity of the cylinder was calculated by subtracting Wa from Wb, and then dividing the result by the volume of the cylinder.

### Statistical analysis

All The experiments were performed in triplicate. All statistical analyses were performed using SPSS version 20 (IBM Corp., Armonk, NY, USA) on a Microsoft Windows-based computer. The vancomycin elution profiles and antibacterial activities presented as ZOI of NAC+/- vancomycin groups were compared to those of vancomycin only groups with paired samples t-test. Statistical significance was set at a *p* < 0.05.

## Results

### NAC/vancomycin elution profile

**Fig. 1:**
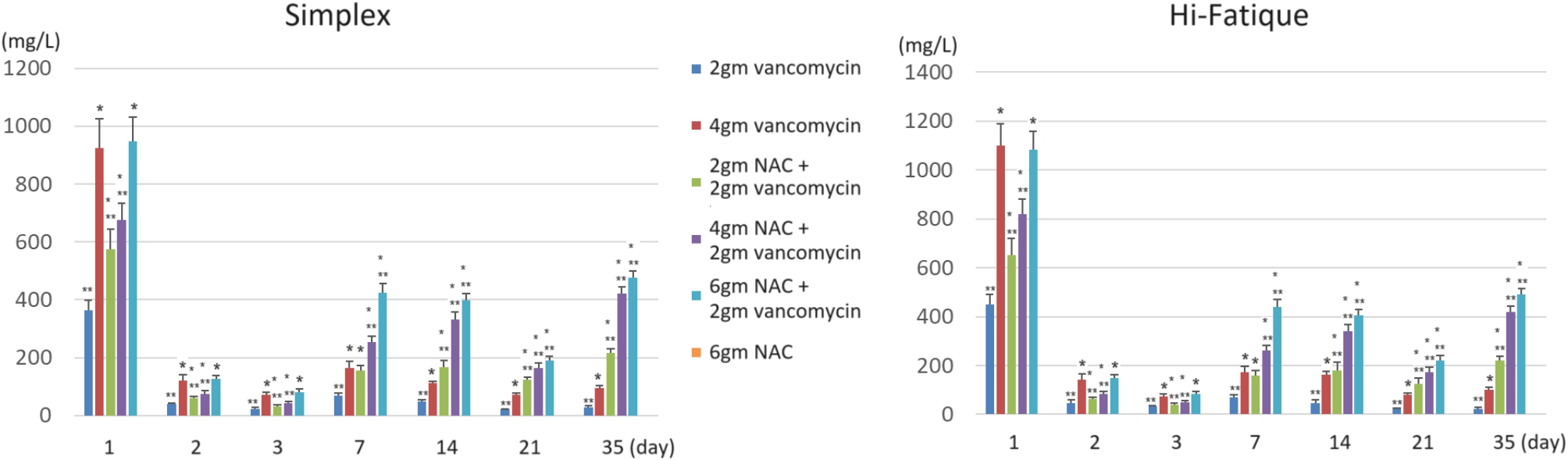
Elution profiles of vancomycin. *P < 0.05, compared to 2gm vancomycin. ** *P* < 0.05, compared to 4gm vancomycin. After day 14, the vancomycin release in the “combination of NAC and vancomycin groups” was significantly higher than in the “vancomycin-only groups”

**Fig. 2:**
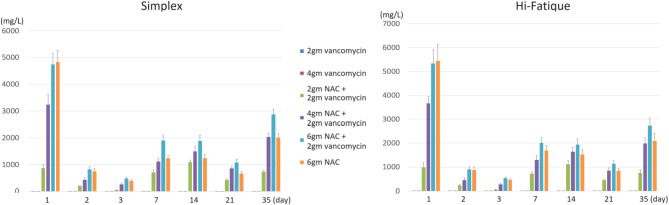
Elution profiles of NAC. The NAC release was positively correlated with the amount of NAC added. Additionally, the simultaneous incorporation of NAC and vancomycin further increased NAC release

**Fig. 3:**
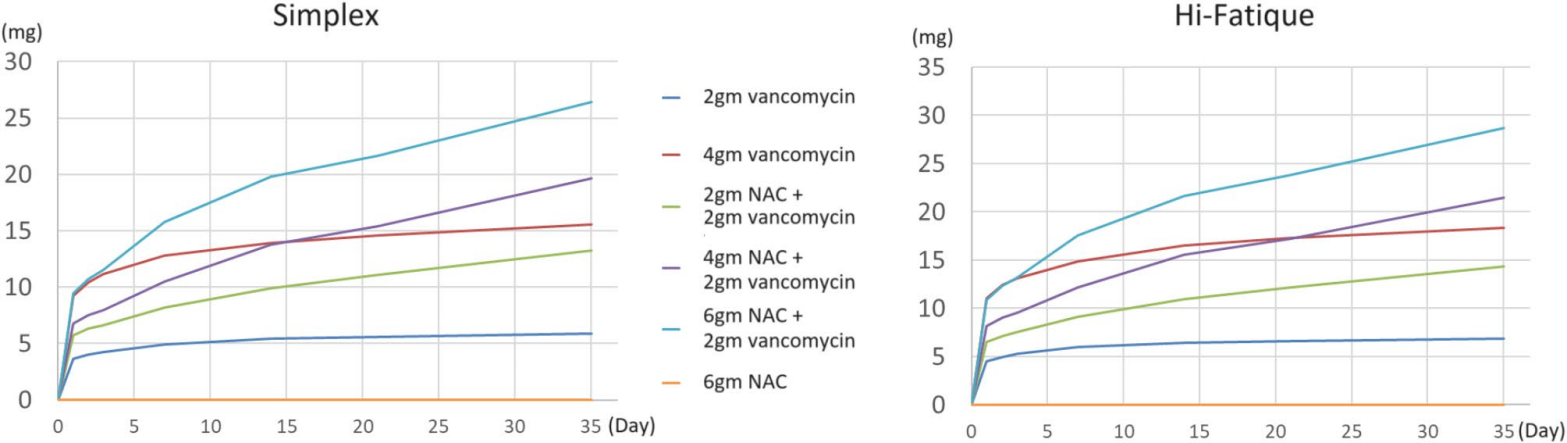
Cumulative vancomycin release. The total vancomycin release in the “4 g NAC + 2 g vancomycin” and “6 g NAC + 2 g vancomycin” groups both exceeded that of the “4 g vancomycin” group

The elution profiles of vancomycin and NAC are shown in Figs. 1 and 2. In general, the addition of NAC increases the release of vancomycin. After the seventh day, adding 2gm, 4gm, and 6gm of NAC to the bone cement containing 2gm vancomycin resulted in a higher release of vancomycin than bone cement containing 4gm of vancomycin alone. The cumulative vancomycin release is shown in Fig. 3. Adding 4gm and 6gm of NAC to the bone cement containing 2gm vancomycin resulted a higher cumulative amount of vancomycin than bone cement containing 4gm of vancomycin alone.

### In vitro cytotoxicity assay

**Fig. 4:**
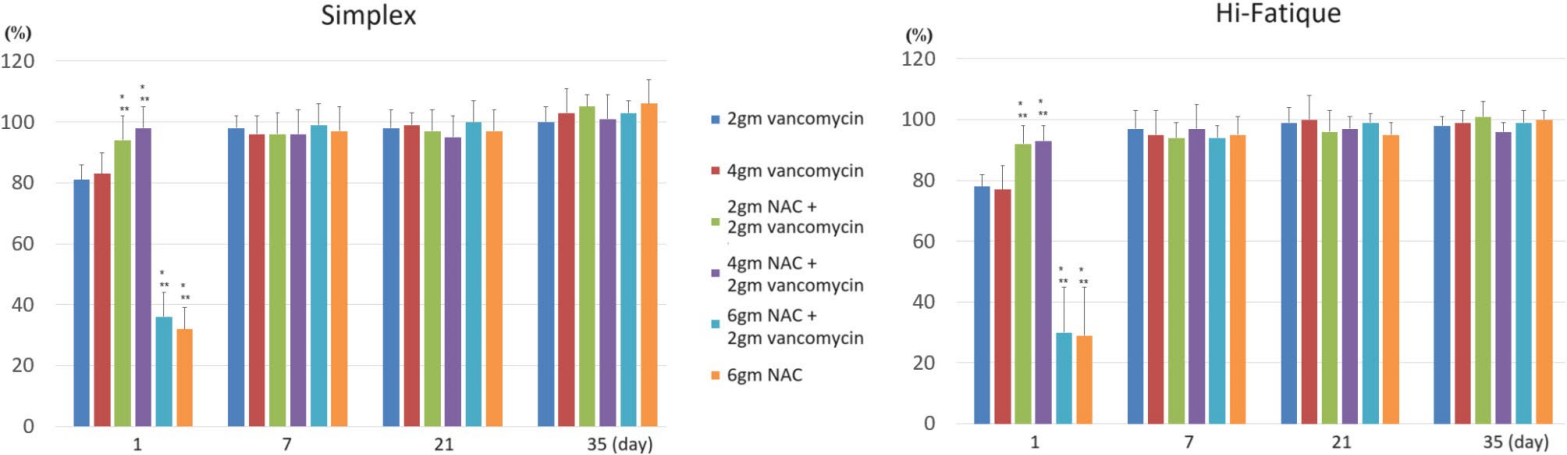
Cell viability evaluated with CKK-8 assay *P < 0.05, compared to 2gm vancomycin. ** *P* < 0.05, compared to 4gm vancomycin. On the first day, adding 2–4 g NAC improved cell viability, whereas adding 6 g NAC reduced cell viability

The results of cell viability are shown in Fig. 4. Except for the first day, cell viability was comparable among all groups. Adding 2gm and 4gm of NAC can increase cell viability, but adding 6gm of NAC significantly decreases cell viability for the first day.

### Bioassay of antibacterial activity

**Fig. 5:**
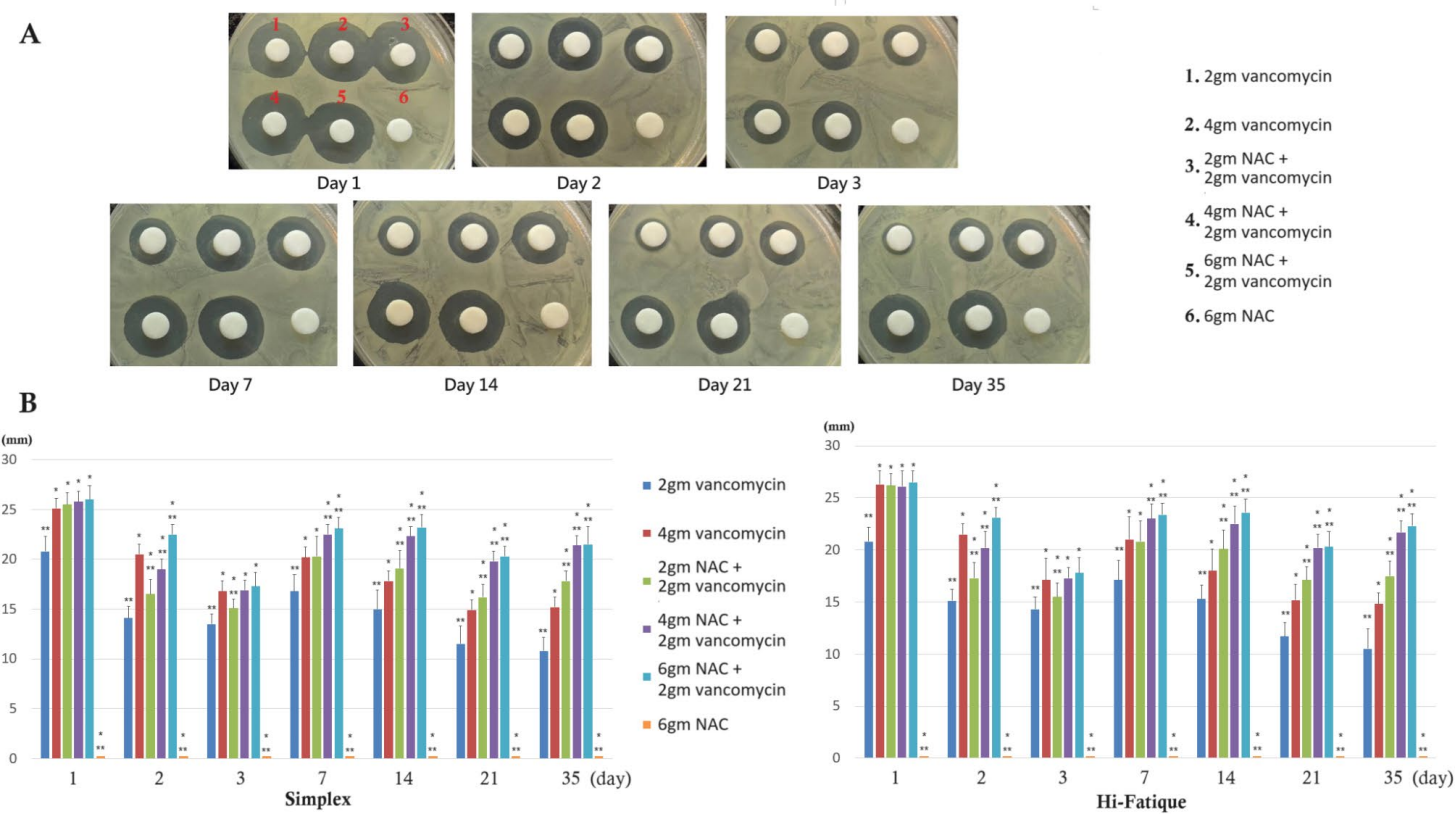
Anti-bacterial activity against MSSA (**A**) The representative photo (**B**) ZOI presentation. *P < 0.05, compared to 2gm vancomycin. ** *P* < 0.05, compared to 4gm vancomycin. After day 14, the “Combination of NAC and vancomycin groups” exhibited significantly greater antibacterial activity than the” vancomycin-only groups”

**Fig. 6:**
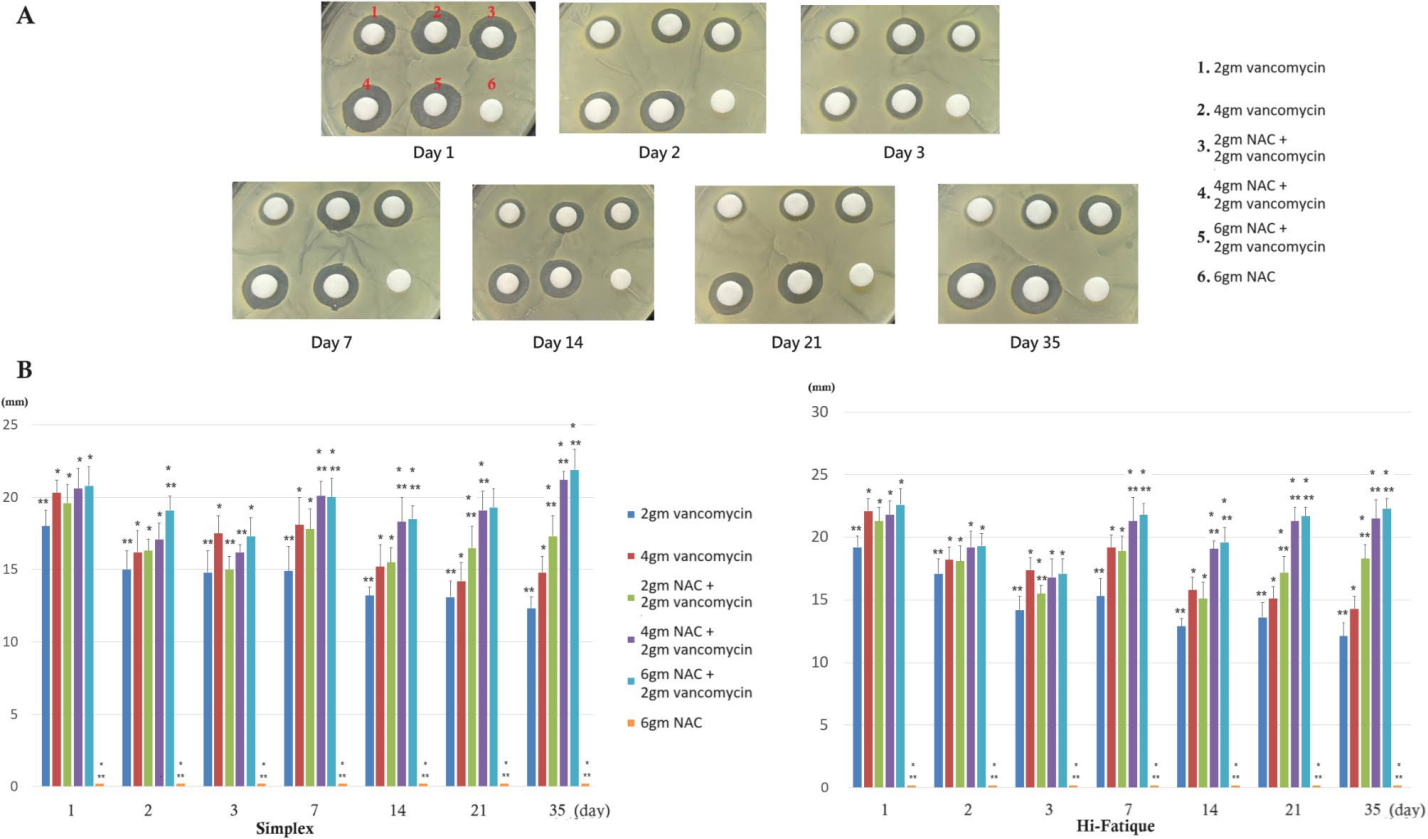
Anti-bacterial activity against MRSA (**A**) The representative photo (**B**) ZOI presentation. *P < 0.05, compared to 2gm vancomycin. ** *P* < 0.05, compared to 4gm vancomycin. After day 14, the “Combination of NAC and vancomycin groups” exhibited significantly greater antibacterial activity than the” vancomycin-only groups”

The antimicrobial activities of the cement-immersed PBS are shown in Figs. 5 and 6. In general, the ZOI of vancomycin alone group significantly decreased over time, while the ZOI of NAC + vancomycin group kept relatively stable. After the seventh day, adding 2gm, 4gm, and 6gm of NAC to the bone cement containing 2gm vancomycin resulted in larger ZOI than bone cement containing 4gm of vancomycin alone. The ZOI of NAC alone group was 0 at any time point.

### Evaluation of porosity of cement cylinders

**Fig. 7:**
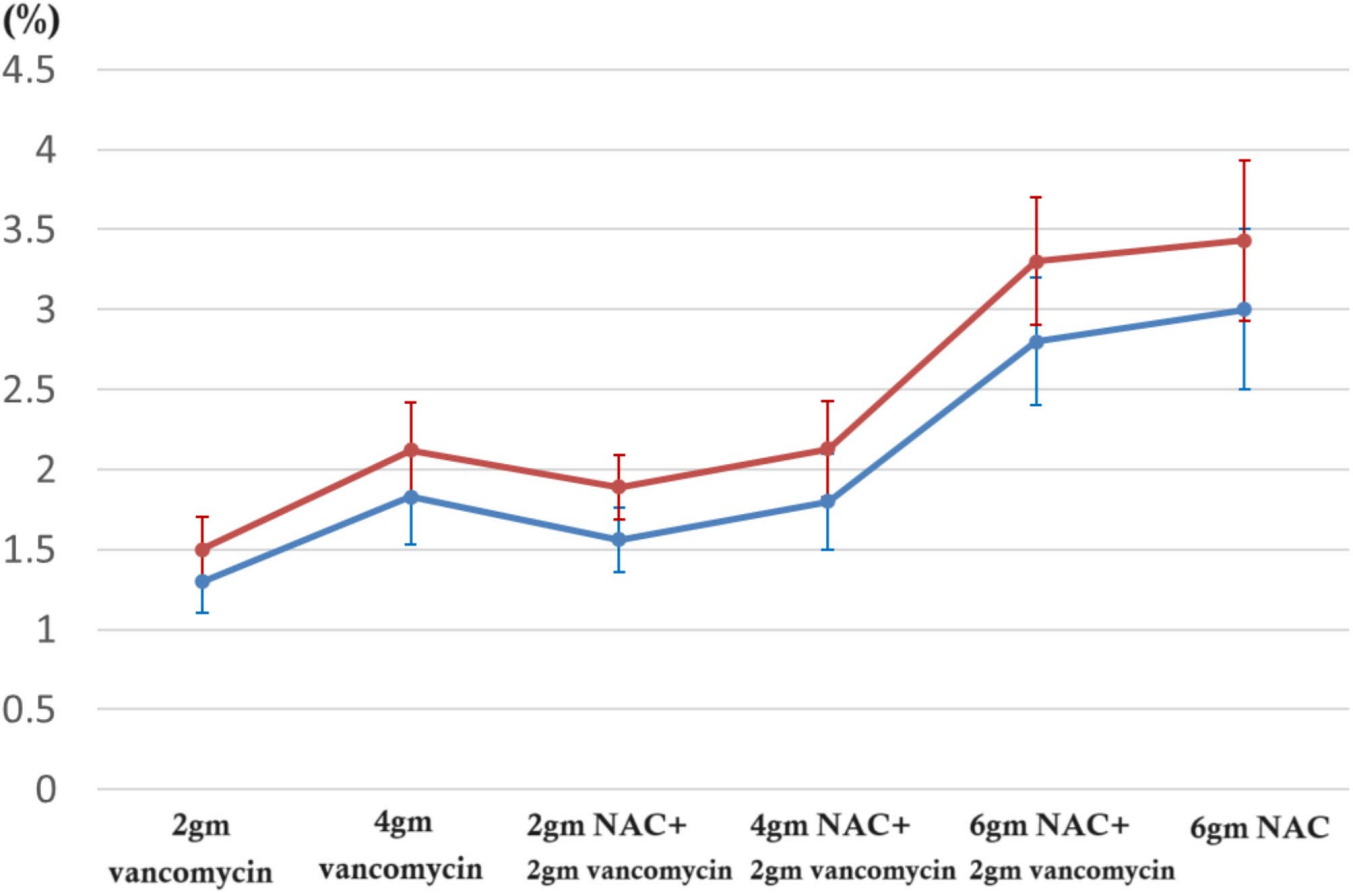
The porosity evaluated with fluid displacement method. The groups with higher NAC content exhibited greater porosity compared to the corresponding vancomycin-only groups

The porosity evaluated with fluid displacement method is shown in Fig. 7. In general, the addition of NAC increased the porosity of bone cement.

## Discussion

The most important finding of this study is that adding NAC and vancomycin simultaneously to PMMA bone cement can extend the antibacterial duration of ALBC. This is likely achieved through increased porosity, which enhances vancomycin release in the later stages of treatment, especially evident after day 7. Interestingly, even with a lower vancomycin dose (2gm), the antibacterial efficacy after 7 days is higher compared to the bone cement with 4gm of vancomycin alone. Theoretically, the advantage of using lower dose antibiotics is that it can prevent systemic toxicity caused by the burst release of high-dose antibiotics at the beginning [22]. Moreover, if the NAC dosage is appropriate, it can reduce the cytotoxicity of the bone cement. Based on the results of this study, the recommended dosage is 2gm vancomycin + 2gm or 4gm NAC in 40gm PMMA. Another important finding is that, unlike the previous study [15], the addition of NAC alone did not show antibacterial properties in this study, and it is not recommended for the treatment of PJI.

During the polymerization process of MMA, free radicals are generated [10], leading to cytotoxicity that induces osteoblast death and promotes osteolysis. Previous studies have demonstrated that incorporating NAC into PMMA bone cement can mitigate its cytotoxic effects [10, 11, 16, 23]. Glutathione, a cysteine derivative primarily located in the cell membrane, plays a critical role in maintaining cellular redox balance. Its redox cycle serves as a key mechanism for neutralizing both exogenous and endogenous free radicals [24, 25]. NAC, the N-acetyl derivative of cysteine, is a well-known antioxidant. Upon deacetylation, NAC releases cysteine, a key precursor in glutathione biosynthesis, thereby enhancing the cellular glutathione system [26]. Additionally, NAC directly scavenges free radicals by donating a hydrogen atom from its thiol group, effectively neutralizing oxidative species [27]. The generation of these free radicals occurs mainly during polymerization, which is in the early stages of treating PJI. Therefore, in our study, the appropriate addition of NAC indeed increases cell survival rates in the early stages.

Our study demonstrates that excessively high concentrations of NAC can induce cell death, aligning with previous reports that associate NAC overdose with severe adverse effects. To date, reports on NAC overdose have been limited to case studies [28–30], documenting complications such as hemolytic uremic syndrome, seizures, cerebral edema, and brain herniation. However, the precise mechanisms underlying NAC-induced cytotoxicity remain unexplored. In this study, NAC release from PMMA showed that groups containing 6 gm NAC alone or in combination with 6 gm NAC and 2 gm vancomycin exhibited significantly higher release levels on the first day compared to other groups. This elevated concentration likely exceeds the drug’s safe threshold, resulting in cytotoxic effects on surrounding cells. Therefore, the addition of 6 gm NAC to bone cement is not recommended.

Although NAC is not an antibiotic, many dental studies have shown its ability to combat multispecies endodontic bacterial biofilms [17, 31, 32]. In orthopedic research, adding NAC alone to bone cement has even been reported to combat the planktonic and biofilm forms of Staphylococcus aureus and Escherichia coli for at least one week, suggesting its potential for treating periprosthetic joint infections [15]. However, our results indicate that bone cement with NAC alone showed a ZOI of 0 for both MSSA and MRSA, clearly demonstrating that this approach cannot replace ALBC and is not recommended for treating bone and joint infections.

Antibiotic release from ALBC has been previously reported in many articles [12, 33]. Although high-dose ALBC is currently a common approach, its drug release pattern is not ideal [33]. When antibiotics are released, there is a burst release on the first day, followed by a sharp decline to very low levels. A prior clinical study revealed that in 40% of patients, the in vivo concentration of vancomycin released from Simplex P cement spacers dropped to undetectable levels by the 7th day post-implantation [34]. This release pattern presents two key issues: the initial burst may lead to vancomycin-induced nephrotoxicity, posing a risk to frail patients [22, 35], and even in those with normal kidney function, the incidence of acute kidney injury is approximately 7% [22]. Meanwhile, the low antibiotic levels in later stages may contribute to treatment failure.

To address these limitations, various studies have explored adding biodegradable poragens, such as α-tricalcium phosphate [36], calcium phosphate [37], PLGA [38], and nanotechnology-based carriers [39]. Although these studies significantly improve the elution profile, these poragens are not yet widely used in clinical applications. Our study found that NAC produces a similar effect, prolonging vancomycin release and maintaining the antibacterial efficacy of ALBC. A possible mechanism for this phenomenon is that the incorporation of NAC increases the porosity of PMMA cement compared to cement containing the same dose of vancomycin alone. As NAC is co-released, the higher porosity facilitates more effective vancomycin release. Similar findings have been reported in previous studies, where the simultaneous incorporation of two different antibiotics influenced their individual release profiles [9, 40, 41]. For example, the co-release of gentamicin and vancomycin resulted in increased release of both antibiotics [40]. In this study, by the fifth week, NAC-enhanced ALBC still demonstrated significantly better antibacterial efficacy than conventional high-dose ALBC. Additionally, because lower doses of antibiotics can be used, it can reduce the initial burst release of vancomycin, thereby preventing the potential toxicity associated with high antibiotic concentrations. Overall, the simultaneous inclusion of NAC and vancomycin results in a more favorable drug release profile.

One limitation of this study is the lack of mechanical property testing for PMMA cement. Bone cement with mechanical properties comparable to the surrounding cancellous bone may enhance prosthesis longevity. However, since this approach is designed for temporary spacers used between the two stages of revision surgery, which are removed in the second stage, their mechanical strength is of limited significance. Another limitation is that this study was conducted in vitro. Whether the antibiotic release profile and antibacterial efficacy observed in this setting can be replicated in vivo remains uncertain. Future animal studies or clinical trials are necessary to validate these findings.

Additionally, this study specifically examined the effects of NAC on vancomycin-impregnated PMMA cement. In clinical practice, different antibiotics may be incorporated depending on the causative bacteria. Whether NAC exerts similar effects with other antibiotics requires further investigation targeting different antibiotics and bacterial strains. Finally, this study was limited to a 35-day experimental period, which closely approximates the standard six-week treatment duration. However, in more complex bone and joint infections requiring extended treatment, the long-term antibacterial effects of NAC remain unclear and cannot be determined from this study.

In conclusion, our results do not recommend using NAC-loaded PMMA for treating PJI. However, adding NAC and vancomycin simultaneously to PMMA bone cement improves the vancomycin elution profile, allows for the use of lower doses of antibiotics, extends the period of antibacterial activity, and enhances biocompatibility. This method has the potential to replace the currently commonly used high-dose ALBC.

## Data Availability

No datasets were generated or analysed during the current study.

## Abbreviations

ALBC: Antibiotic-loaded bone cement
HPLC: High-performance liquid chromatography
MRSA: Methicillin-resistant Staphylococcus aureus
MSSA: Methicillin-sensitive Staphylococcus aureus
NAC: N-acetylcysteine
PBS: Phosphate-buffered saline
PJI: Prosthetic joint infection
PMMA: Polymethylmethacrylate
ZOI: Zone of inhibition
